# Editorial: 365 days of progress in cancer genetics

**DOI:** 10.3389/fonc.2023.1270902

**Published:** 2023-09-26

**Authors:** Magdalena Ratajska, Claudio Sette, Heather E. Cunliffe

**Affiliations:** ^1^ Department of Pathology, Dunedin School of Medicine, University of Otago, Dunedin, New Zealand; ^2^ Department of Biology and Medical Genetics, Medical University of Gdansk, Gdansk, Poland; ^3^ Department of Neuroscience, Section of Human Anatomy, University of the Sacred Hearth, Rome, Italy; ^4^ Gemelli Science and Technology Park (GSTeP)-Organoids Research Core Facility, Fondazione Policlinico Agostino Gemelli IRCCS, Rome, Italy

**Keywords:** cancer genetics, genomics, neoantigens, immunotherapy, prognosis biomarkers

Progress in cancer research continues to advance rapidly, with significant developments in understanding the genetic basis of cancer, identification of new cancer-associated genes, and the development of targeted therapies based on genetic profiling. Advances are accelerating on many fronts due in large part to increased robustness of high throughput technologies and improvements in biospecimen acquisition and management. Notable key areas of progress in cancer genetics include cancer genomics, liquid biopsies, immunotherapy and biomarkers, precision medicine and cancer risk assessment.

Cancer genomics: Advances in sequencing technologies (including at the single-cell level) have enabled researchers to identify genetic aberrations that drive cancer development and progression (1). Large-scale genomic and transcriptomic studies continue to uncover numerous new cancer-associated genes and molecular pathways, with increasing appreciation for the significance of the impact of intratumoural complexity on disease progression (2). Importantly, it has become a requirement for bioinformatically-derived discoveries to include molecular, functional and/or clinical validation of findings to facilitate novel observations to gain traction in clinical research and enable advances in patient management and prognostication.

Liquid biopsies: Liquid biopsies, which involve analysing tumour-derived DNA and other biomarkers from blood samples, have shown promise in non-invasively detecting cancer, monitoring treatment response, and identifying molecular genetic changes associated with drug resistance (3).

Immunotherapy and biomarkers: Genetic profiling has been crucial in predicting responses to immunotherapies. Identifying specific genetic features of tumours can help determine which patients are more likely to benefit from immune checkpoint inhibitors and other immunotherapies (4).

Precision medicine: Personalized treatment approaches based on the genetic characteristics of an individual’s tumour is becoming mainstream where cancer centre infrastructure and funding permits. Targeted therapeutic approaches to specifically addressing the unique genetic alterations present in a patient’s cancer are leading to improved treatment outcomes and reduced side effects across a broad spectrum of neoplasms (5).

Cancer risk assessment: Genetic testing and screening for inherited cancer predisposition (including syndromes) has become more accessible, allowing for early detection and prevention strategies in individuals with a higher risk of developing certain types of cancer.

This editorial summarises four recent articles highlighting important progress in Cancer Genetics in diverse neoplastic disease contexts. Firstly, Zhang et al. reported an interesting study evaluating an association between two microRNA polymorphisms, miR-671 rs1870238 and miR-671 rs2446065, with susceptibility to soft tissue sarcomas (STSs). STSs are a highly heterogeneous group of tumours, presenting ambiguous clinical and histopathological features making diagnosis and therapy challenging. Notably, at present, there are no useful biomarkers for prevention measures or effective treatment follow-up. It is widely accepted that miRNAs are differentially expressed in STSs, with utility for clinically relevant STS subclassification. Therefore, screening for potential SNPs that might alter miRNAs and their role in the cell is an interesting approach when looking for a predisposition to STSs. Zhang and colleagues genotyped 169 patients diagnosed with different STSs and 170 healthy controls for 17 SNPs in six miRNAs and demonstrated a significant association between miR-671 rs1870238 and miR-671 rs2446065 and the risk of developing STSs. Specifically, rs1870238 (GC/CC) and rs2446065 (CG+GG) had 1.963- and 1.838-fold increased risk of developing STSs. These exciting results indicate a potential role of rs1870238 and rs2446065 in the predisposition to STSs and should be considered for further validation.

Sakai et al. presented a case report of a 12-year-old boy with a phosphaturic mesenchymal tumour (PMT) with a novel fusion gene *NIPBL-BEND2*. PMTs are rare tumours, secreting FGF23, leading to hypophosphatemia and tumour-induced osteomalacia. To date, PMTs have been reported with two different *FN1* gene fusions, *FN1-FGFR1* and *FN1-FGF1*; however, these alterations were present in less than 50% of cases. Therefore, the pathobiology of the remaining PMTs remains unclear. The patient had no history of metabolic bone disease, yet he presented with a deficiency of bone mineralisation similar to that observed in patients with rickets and gait difficulties. On initial biochemical examination, the patient had a markedly high serum FGF23 level, which started decreasing immediately after tumour resection and had normalised 3 hours post surgery. Muscle weakness gradually improved, and gait disturbance normalised two months post surgery. RNA-seq analysis of the resected tumour did not reveal any *FN1* fusions but detected a novel *NIPBL-BEND2* fusion. Interestingly, the *NIPBL-BEND2* fusion gene, when cloned into HEK293T cells (to enable production of recombinant proteins), induced cell proliferation and upregulation of the MYC pathway, suggesting a potential new aetiology of PMT.

The study presented by Li et al. aimed to determine the utility of lncRNA AP004608.1 as an independent predictive marker of survival for patients diagnosed with prostate cancer (PCa).

The authors performed an initial *in silico* analysis using The Cancer Genome Atlas (TCGA) database (https://www.cancer.gov/tcga) and then validated their results using a second, independent dataset (6). Li et al. identified significantly lower expression of AP004608.1 in normal prostatic tissue compared to PCa, and also showed that the level of AP004608.1was an independent predictor of patient overall survival (taking TNM stage into consideration), especially for short follow-up (ROC 0.982 for follow-up up to 12 months). The predictive value of AP004608.1 decreased with extension of the observation time, reaching 0.795 and 0.568 for five and ten years of observation, respectively. These findings are consistent with literature supporting links between abnormal lncRNA expression and PCa prognosis. With PCa being characterized by high disease burden, potential biomarkers that might improve patient management are of great importance, making the result of Li et al. an exciting finding and worthy of further verification.

Finally, Lao et al. investigated genomic alterations and neoantigen characteristics of gastroesophageal tumours (ACGEJ) to identify novel therapeutic targets, a risk model to predict patient survival. Whole exome sequencing was performed on 55 paired samples from ACGEJ patients to identify somatic mutations and copy number aberrations. Findings were compared with their previous RNAseq data, and data available *via* TCGA, to predict neoantigens and to evaluate genes significantly associated with the presence of T-cell infiltrates. Recurrent aberrations were identified in *MAP2K7*, *RNF43*, *RHOA, CCNE1* and *VEGFA* genes, and a distinct neoantigen landscape. In addition, several infiltration-related Hub genes identified by RNAseq. This study provides important new insight for neoantigen-based immunotherapeutic targets for ACGEJ treatment and effective disease prognosis biomarkers.

## Author contributions

MR: Writing – original draft, Writing – review & editing. CS: Writing – review & editing. HC: Writing – review & editing, Writing – original draft.
